# Plasmid Fusion and Recombination Events That Occurred during Conjugation of *poxtA*-Carrying Plasmids in Enterococci

**DOI:** 10.1128/spectrum.01505-21

**Published:** 2022-01-19

**Authors:** Xinxin Shan, Mengyan Yang, Nannan Wang, Stefan Schwarz, Dexi Li, Xiang-Dang Du

**Affiliations:** a College of Veterinary Medicine, Henan Agricultural University, Zhengzhou, People’s Republic of China; b Institute of Microbiology and Epizootics, Centre for Infection Medicine, Department of Veterinary Medicine, Freie Universität Berlin, Berlin, Germany; Forschungszentrum Jülich

**Keywords:** enterococci, linezolid resistance, *poxtA*, plasmid integration

## Abstract

Linezolid plays a crucial role in the treatment of infections caused by multiresistant Gram-positive bacteria. The *poxtA* gene not only confers oxazolidinone and phenicol resistance but also decreases susceptibility to tetracycline. In this study, we investigated structural changes in mobilizable *poxtA*-carrying plasmids in enterococci which occurred during conjugation experiments using S1-PFGE (pulsed-field gel electrophoresis), Southern blot hybridization, and whole-genome sequencing (WGS) analysis. Two *poxtA*-carrying strains were identified in Enterococcus faecalis E006 and Enterococcus lactis E843, respectively. E. faecalis E006 contains the 121,520-bp conjugative plasmid pE006-121 and the 19,832-bp mobilizable *poxtA*-carrying plasmid pE006-19, while *E. lactis* E843 contains the 171,930-bp conjugative plasmid pE843-171 and the 27,847-bp mobilizable *poxtA*-carrying plasmid pE843-27. Moreover, both *poxtA*-carrying plasmids were mobilized by their respective conjugative plasmid in enterococci by plasmid fusion; one was generated by homologous recombination in E. faecalis through an identical 864-bp homologous region in the plasmids of the parental strain, while another was generated by an IS*1216E*-mediated plasmid integration in *E. lactis*, involving a replicative transposition.

**IMPORTANCE** Until now, all the *poxtA* genes described in enterococci, including E. faecalis, E. faecium, and E. hirae, are plasmid-borne, suggesting that plasmids play an important role in the dissemination of the *poxtA* gene among enterococci. This study showed that the mobilizable *poxtA*-carrying plasmid could transfer with the help of conjugative plasmid in enterococci via plasmid fusion, with one generated by homologous recombination in E. faecalis, and another by replicative transposition in *E. lactis*. During both the fusion events, the *poxtA*-carrying plasmids changed from nonconjugative to conjugative, leading to the generation and enhanced dissemination of the larger phenicol-oxazolidinone-tetracycline resistance-encoding plasmids in enterococci.

## INTRODUCTION

Linezolid is a one of the last therapeutic options for the treatment of clinical infections caused by MDR Gram-positive bacteria, including methicillin-resistant Staphylococcus aureus (MRSA), vancomycin-resistant enterococci (VRE) and penicillin-resistant Streptococcus pneumoniae (PRSP). Florfenicol is a broad-spectrum phenicol drug exclusively used in animals to control respiratory tract infections (1, 2). However, the emergence of cross-resistance to linezolid and florfenicol poses a serious challenge to both human and veterinary medicine. To date, at least seven acquired linezolid/florfenicol resistance genes, belonging to three different groups, have been reported, including *cfr*, *cfr*(B), *cfr*(C), *cfr*(D), *cfr*(E), *optrA*, and *poxtA* (3–10). The *cfr* gene and its variants code for 23S rRNA methylases, while the *optrA* and *poxtA* genes encode the ribosome-protective proteins of the ABC-F family. The *cfr* gene confers resistance to phenicols, lincosamides, oxazolidinones (linezolid but not tedizolid), pleuromutilins, and streptogramin A (the so-called PhLOPSA phenotype). It was first described in Staphylococcus sciuri (recently reclassified as *Mammaliicoccus sciuri*) and thereafter detected in various Gram-positive and Gram-negative bacteria. Unlike *cfr*, the *optrA* gene confers resistance to tedizolid, a novel oxazolidinone, in addition to linezolid and phenicol resistance. It was first identified in Enterococcus faecalis and E. faecium (4) but has also been detected in various other Gram-positive bacteria (11). The *poxtA* gene, which confers decreased susceptibility to tetracycline in addition to phenicol and oxazolidinone resistance, has recently been identified in MRSA and enterococci (9–14). More recently, *poxtA2* was detected in Enterococcus gallinarum (15).

The prevalence of the *poxtA* gene has been investigated in enterococci from food-producing animal farms in China, Italy, and Korea, as well as in linezolid-resistant *Enterococcus* spp. (LRE) from patients in Ireland, Spain, and France (13, 16–22). In China, Lei et al. reported that the *poxtA* gene was present in 5.5% (19/345) of enterococci isolated from 36 food-producing animal farms across 12 provinces in 2017 (16). However, in our previous study, the *poxtA* gene was present in 57.9% (66/114) of florfenicol-resistant enterococci isolated from two swine farms in Henan Province in 2018, possibly the result of widespread use of florfenicol and doxycycline in these swine farms (13). In Italy, Fioriti et al. reported the *poxtA* gene with the prevalence of 20.7% (30/145) in florfenicol-resistant enterococci isolated from swine fecal samples (17). In Korea, 156 linezolid-resistant isolates were detected in 5,482 enterococci isolated from food-producing animals during 2008 to 2018, and 23.1% of these (36/156) had the *poxtA* gene (18). In addition, Kim et al. reported the *poxtA* gene with a prevalence of 2.5% (8/327) in enterococci isolated from food-producing animals and meat in Korea in 2018 (19). In Ireland, 15 *poxtA*-carrying enterococci were identified in 154 LRE which were recovered from patients in 14 hospitals between June 2016 and August 2019 (20). In Spain, clinical LRE isolates from different hospitals were submitted to the Spanish Reference Laboratory from 2015 to 2018, and 6.2% of them (6/97) were *poxtA*-positive (21). In France, out of 466 LRE received by the National Reference Centre for Enterococci from French hospitals between 2016 and 2020, 47 (10.1%) were *poxtA*-positive (22).

Previously, all of the *poxtA* genes described in enterococci have been plasmid-borne, suggesting that plasmids play an important role in the dissemination of the *poxtA* gene among enterococci (22, 23). In this study, two mobilizable *poxtA*-carrying plasmids were identified in E. faecalis and *E. lactis*, respectively. In addition, two fusion events involving homologous recombination and replicative transposition were investigated, which transferred the *poxtA* gene from mobilizable plasmids into conjugative plasmids. Consequently, continuous monitoring of *poxtA*-mediated linezolid resistance in enterococci is urgently needed.

## RESULTS

### Linezolid resistance mediated by plasmids in enterococci was transferable.

**FIG 1:**
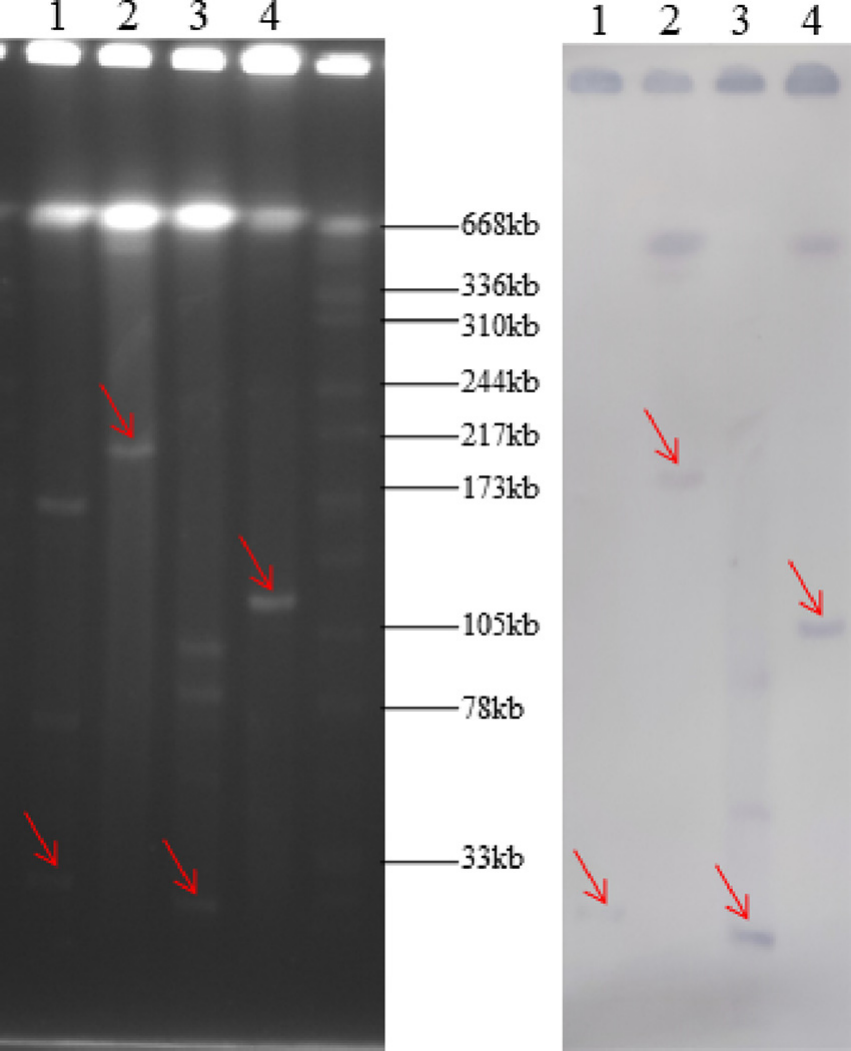
Detection of *poxtA*-carrying plasmids in E. faecalis E006, *E. lactis* E843 and their transconjugants by S1-PFGE (left) and Southern hybridization (right) with *poxtA* specific probe. Lane 1, *E. lactis* E843; lane 2, transconjugant E. faecium E843×GE-1-TC1; lane 3, E. faecalis E006; lane 4, transconjugant E. faecalis E006×JH2-2-TC1.

The florfenicol- and linezolid-resistant E. faecalis E006 (ST692) and *E. lactis* E843 were *poxtA*-positive. Conjugation assays showed that the *poxtA* gene in E. faecalis E006 and *E. lactis* E843 could be transferred to the recipient strains E. faecalis JH2-2 (ST8) and E. faecium GE-1 (ST515), respectively. The corresponding transconjugants were designated E. faecalis E006×JH2-2-TC1 (ST8) and E. faecium E843×GE-1-TC1 (ST515), respectively. Antimicrobial susceptibility testing (AST) results for the donor strains, their transconjugants, and the recipient strains are shown in Table 1. Both transconjugants displayed elevated MICs of the respective antimicrobial agents compared with those of the recipient strain. S1-PFGE (pulsed-field gel electrophoresis) showed that there was a single plasmid in both the transconjugant E. faecalis E006×JH2-2-TC1 and E. faecium E843×GE-1-TC1, but two plasmids in their parental strains (Fig. 1). Moreover, the size of the plasmids in the transconjugants was almost equivalent to that of one of the plasmids in their parental strains. Southern blot hybridization confirmed that the *poxtA* gene was located on the smaller plasmid in the parental strains and on the single plasmid in the transconjugants (Fig. 1). To investigate how the *poxtA* gene could be transferred from the small plasmid in the parental strain to the larger plasmid in the transconjugants, whole-genome DNA sequencing was performed for all four stains (E. faecalis E006, *E. lactis* E843, transconjugants E. faecalis E006×JH2-2-TC1, and E. faecium E843×GE-1-TC1). In addition, the larger plasmids in E. faecalis E006×JH2-2-TC1 and E. faecium E843×GE-1-TC1 could further be transferred to the recipient strains E. faecalis E533 (ST69) and E. faecalis JH2-2 at frequencies of 1.7 × 10^−9^ and 1.4 × 10^−6^, respectively, indicating that the *poxtA-*carrying plasmids can change from mobilizable to conjugative and become self-transferable.

**TABLE 1:** The antimicrobial susceptibilities of the donor strains, transconjugants, and recipient strains used in this study^*a*^

Strains	MICs (mg/L)									
	RIF	FUS	FFC	LZD	TZD	TET	GEN	VAL	LIN	ERY
E. faecalis										
Donor E006	<1	2	128	16	4	128	>128	>128	>128	>128
Recipient JH2-2	>128	>128	2	2	1	2	32	>128	16	<1
Transconjugant E006×JH2-2-TC1	>128	>128	64	16	4	<1	>128	>128	>128	>128
E. faecium										
Donor E843	16	2	128	4	1	128	>128	32	>128	>128
Recipient GE-1	>128	>128	1	1	0.5	64	8	<1	16	2
Transconjugant E843×GE-1-TC1	>128	128	8	4	1	128	>128	16	>128	>128

a RIF, rifampicin; FUS, fusidic acid; FFC, florfenicol; LZD, linezolid; TZD, tedizolid; TET, tetracycline; GEN, gentamicin; VAL, valnemulin; LIN, lincomycin; ERY, erythromycin.

### Genetic basis of transmission of linezolid resistance in *E. faecalis*.

**FIG 2:**
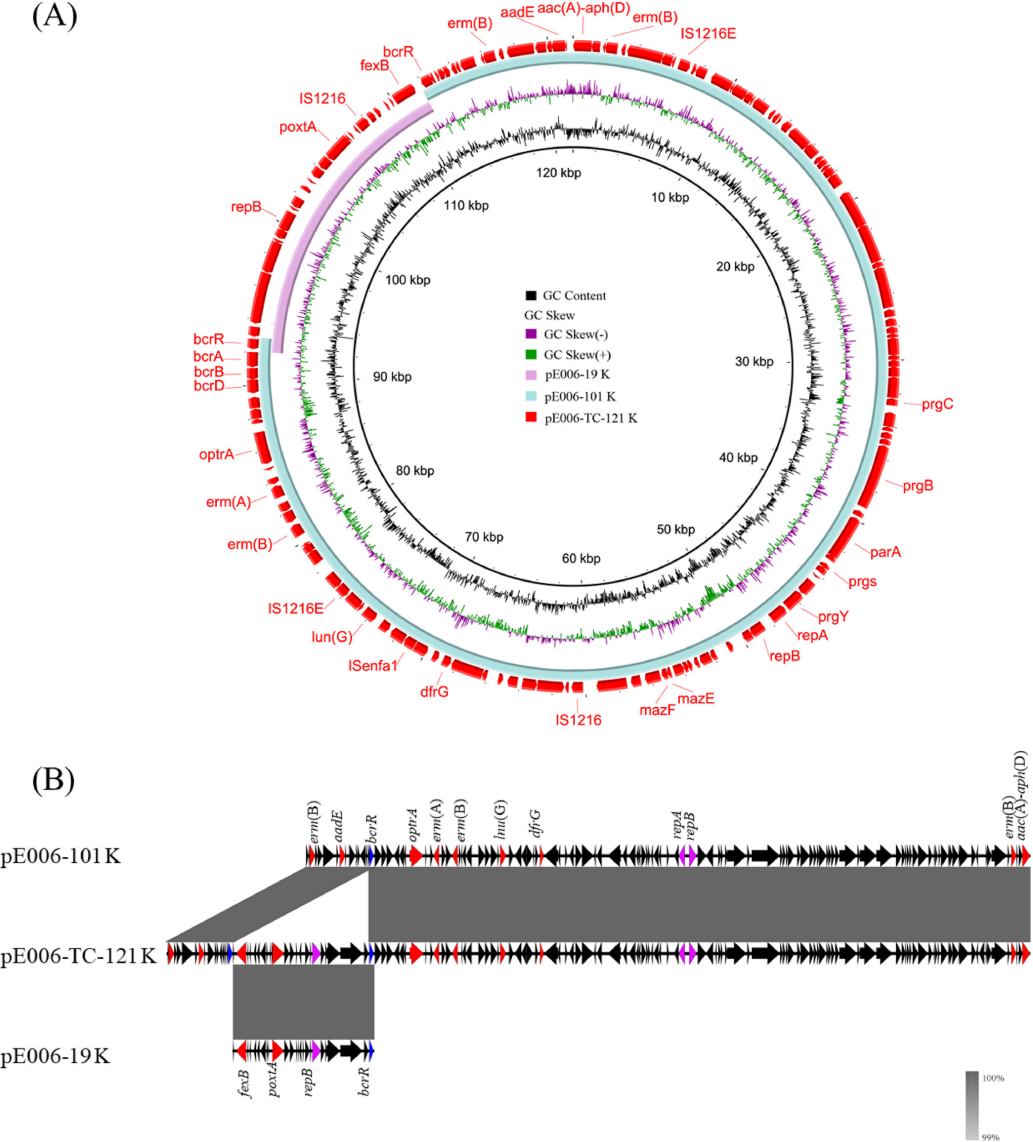
Formation of conjugative fusion plasmid pE006-TC-121 co-carrying *optrA* and *poxtA*. Alignment of *poxtA*-carrying mobilizable plasmid pE006-19 and pheromone responsive conjugative plasmid pE006-101 recovered from E. faecalis E006 using the BLAST Ring Image Generator (BRIG) (panel A) and Easyfig (panel B).

For the two plasmids in E. faecalis E006, the larger plasmid was designated pE006-101 and the smaller one was designated pE006-19. The conjugative plasmid pE006-101 is 101,692 bp in size and harbors three copies of the macrolide-lincosamide-streptogramin B resistance gene *erm*(B) and one copy of *erm*(A). In addition, it also carries single copies of the florfenicol/oxazolidinone resistance gene *optrA*, the lincosamide resistance gene *lnu*(B), the trimethoprim resistance gene *dfrG*, the gentamicin-tobramycin-kanamycin resistance gene *aac*(A)*-aph*(D), the streptomycin resistance gene *aadE*, and the bacitracin resistance operon *bcr*ABDR. Moreover, like the previously described *optrA*-carrying pheromone responsive plasmid pEF10748, pE006-101 is a *rep9*–type plasmid (pCF10 prototype, the best-studied pheromone responsive plasmid) by plasmid typing, and harbors the essential sex pheromone response genes, such as *prgA*, *prgB*, and *prgC* (Fig. 2A; 24). The mobilizable plasmid pE006-19 is 19,832 bp in size and carries the florfenicol resistance gene *fexB*, the gene *poxtA*, and *bcrR*, which is a part of the bacitracin resistance operon *bcr*ABDR (Fig. 2A).

**FIG 3:**
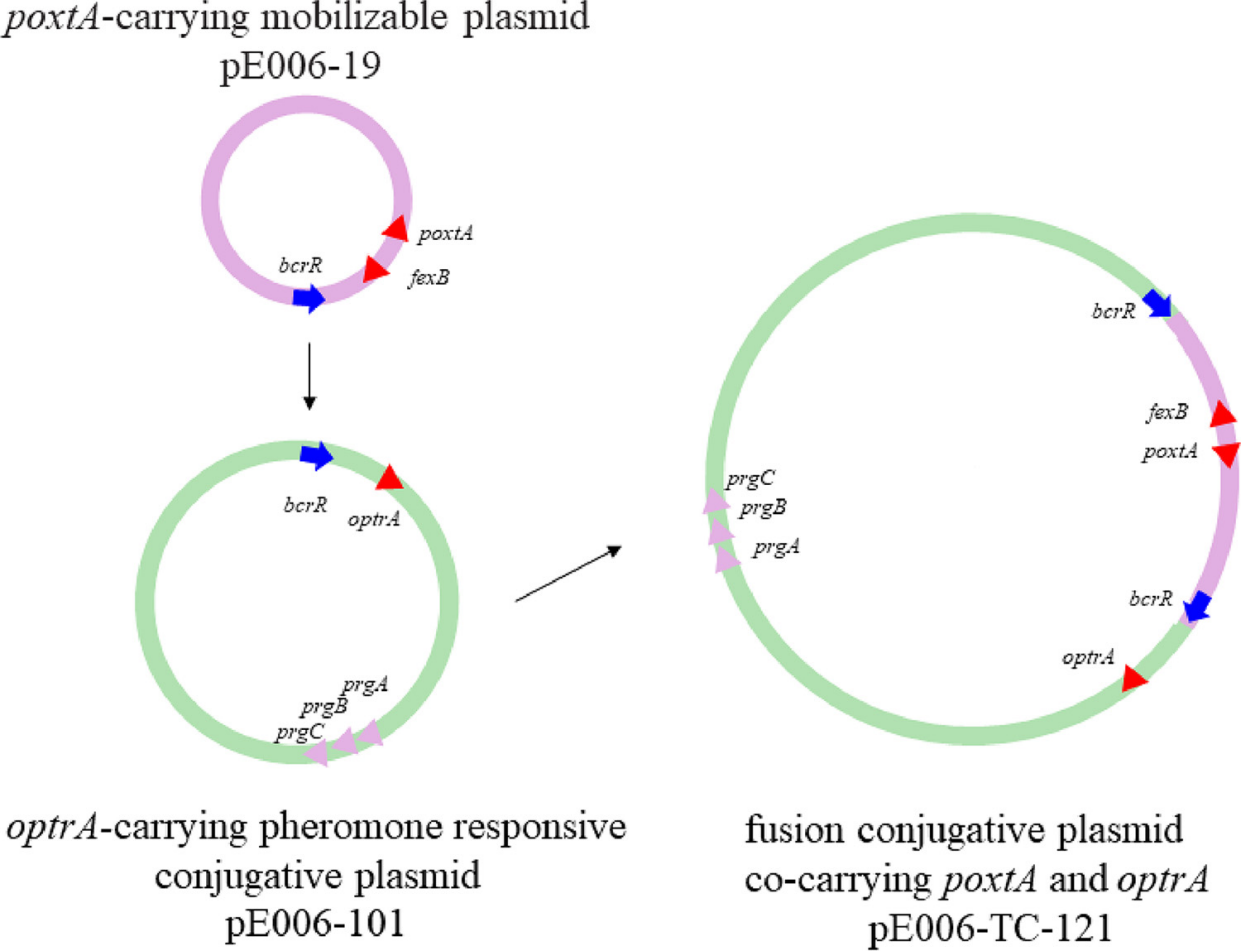
Mechanism of plasmid fusion by homologous recombination. Fusion plasmid was generated by homologous recombination involving an 864-bp HR which included *bcrR*.

Only one conjugative plasmid, 121,537 bp in size and designated pE006-TC-121, was detected in the transconjugant E006×JH2-2-TC1. Sequence analysis revealed that pE006-TC-121 was obtained through fusion between pE006-101 and pE006-19 (Fig. 2). Detailed analysis of these three plasmids suggested that the mechanism of plasmid fusion was due to homologous recombination involving an 864-bp homologous region (HR) which included *bcrR*. One copy each of this HR was detected in pE006-101 and pE006-19, but two copies were detected in pE006-TC-121. The HRs in all three plasmids were identical. According to these results, we propose that pE006-101 and pE006-19 underwent homologous recombination at the HR, resulting in the formation of the fusion plasmid pE006-TC-121 (Fig. 3).

### Genetic basis of transmission of linezolid resistance in *E. lactis*.

**FIG 4:**
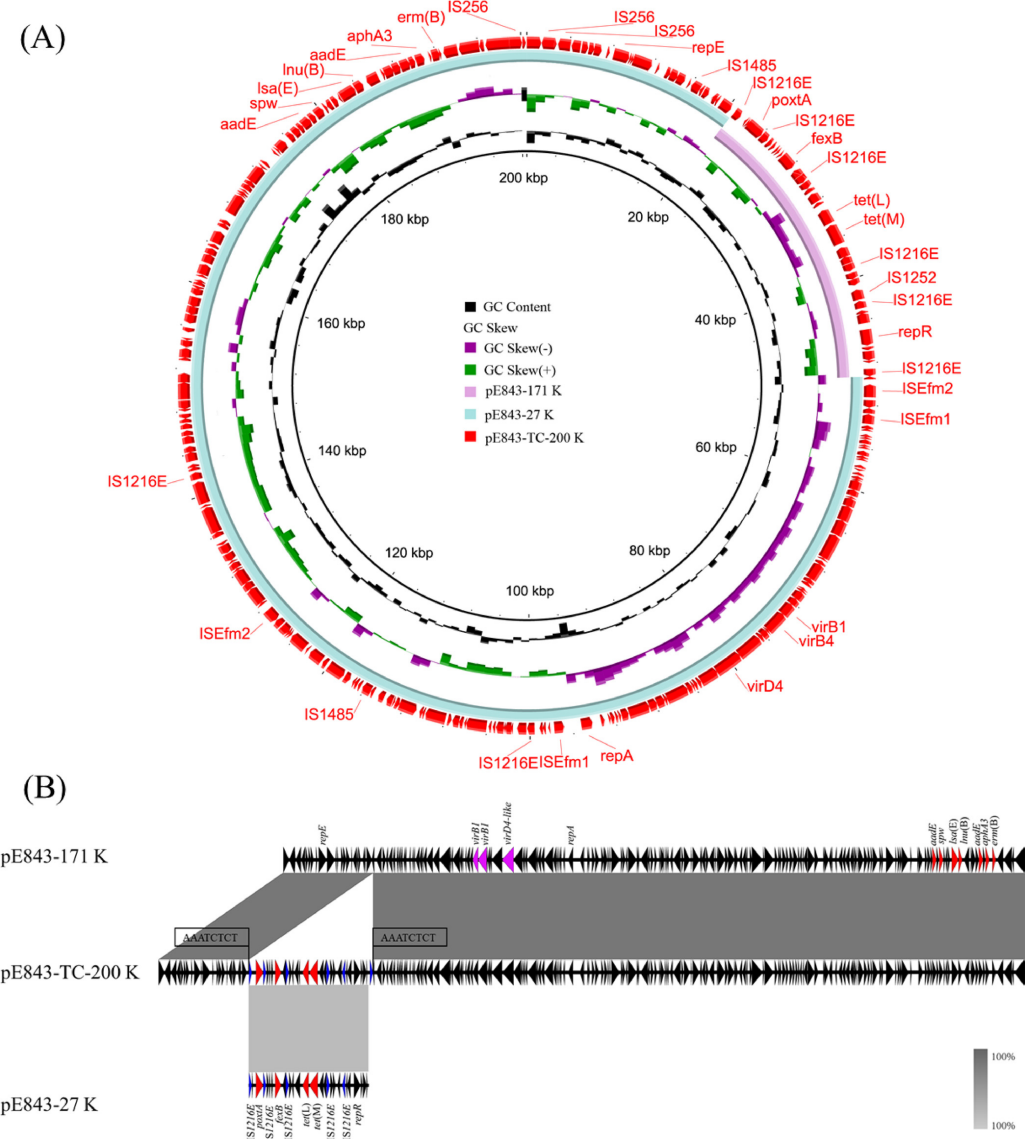
Formation of conjugative fusion plasmid pE843-TC-200. Alignment of *poxtA-*carrying mobilizable plasmid pE843-27 and conjugative pLG1-like plasmid pE843-171 recovered from *E. lactis* E843 using the BLAST Ring Image Generator (BRIG) (panel A) and Easyfig (panel B).

A plasmid fusion event was also observed in *E. lactis* E843. There are two plasmids in the parental strain, designated pE843-171 and pE843-27, as well as one plasmid in the transconjugant E843×GE-1-TC1, designated pE843-200. Plasmid pE843-171 is 171,930 bp in size and harbors two copies of the streptomycin resistance gene *aadE*, as well as single copies of the macrolide-lincosamide-streptogramin B resistance gene *erm*(B), the neomycin-kanamycin resistance gene *aphA3*, the lincosamide resistance gene *lnu*(B), the pleuromutilin-lincosamide-streptogramin A (PLS_A_) resistance gene *lsa*(E), and the spectinomycin resistance gene *spw*. All resistance genes were clustered in an ∼14-kb region. pE843-171 was found to be a pLG1-like conjugative plasmid with essential type IV secretion system (T4SS) genes (25), such as *virB1*, *virB4*, and *virD4* homologues (Fig. 4A). The mobilizable plasmid pE843-27 is 27,847 bp in size and carries the florfenicol resistance genes *poxtA* and *fexB* and the tetracycline resistance genes *tet*(M) and *tet*(L). In pE843-27, there are a total of six copies of IS*1216E*, with five of them located in the same orientation (Fig. 4A).

**FIG 5:**
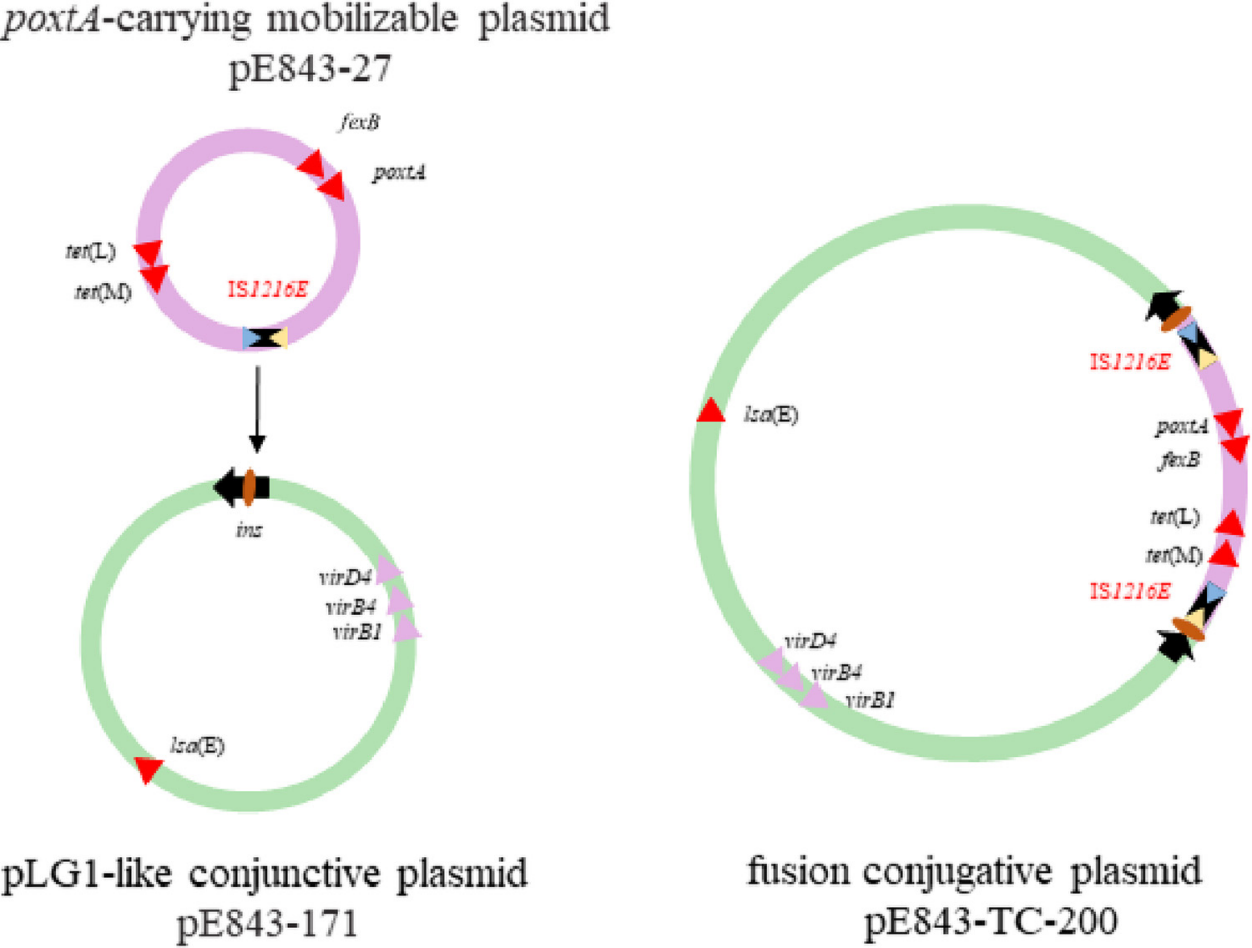
Mechanism of plasmid fusion by replicative transposition. IS*1216E* in mobilizable plasmid pE843-27, attached to the target site (AAATCTCT) in the pLG1-like plasmid pE843-171, forming a characteristic 8-bp target site duplication (TSD, AAATCTCT) and generating an additional copy of IS*1216E* downstream from the fusion plasmid pE843-200.

As in E006×JH2-2-TC1, there was only one conjugative plasmid in the transconjugant E843×GE-1-TC1. This plasmid, designated pE843-TC-200, is 200,566 bp in size (Fig. 4A). Based on the detailed sequence analysis, the formation of this fusion plasmid was due to a series of genetic events generated by IS*1216E*. We propose that IS*1216E* in the mobilizable plasmid pE843-27 attached to the target site (AAATCTCT) in the pLG1-like plasmid pE843-171; then, the replicative transposition occurred, and pE843-27 was inserted into pE843-171, forming a characteristic 8-bp target site duplication (TSD, 5′-AAATCTCT-3′) and generating an additional copy of IS*1216E* in the fusion plasmid pE843-200 (Fig. 4 and 5).

## DISCUSSION

Enterococci are opportunistic pathogens which have become one of the main causes of nosocomial and community-acquired human infections, including septicemia, endocarditis, and urinary tract infections (26). Their intrinsic resistances and ability to acquire resistances through mobile genetic elements (MGEs) have compromised the choice of therapeutic options to treat enterococcal infections. Among MGEs, conjugative plasmids play a key role in the dissemination of resistance genes, as conjugative plasmids are self-transmissible and can mobilize nonconjugative plasmids present in the same enterococcal cell (27).

In Gram-negative bacteria, IS*26*-mediated replicative transposition and translocation play an important role in mobilizing antimicrobial resistance genes (28, 29). IS*1216* in Gram-positive bacteria and IS*26* in Gram-negative bacteria belong to the IS*6*/IS*26* family of bacterial insertion sequences and are assumed to exert similar functions. To our knowledge, the genetic environment of the *poxtA* gene, as described previously, contains IS*1216E* (23), suggesting its potential role in the dissemination of the *poxtA* gene. However, how IS*1216E* promotes the transmission of the *poxtA* gene is not yet fully explored.

In our previous study, the role of IS*1216E*-mediated transposition and translocation was shown in the formation of *poxtA* fusion plasmids (30). By IS*1216E-*mediated replicative transposition, a fusion *poxtA*-carrying plasmid was generated through a *poxtA*-carrying transposon fused into another small plasmid. Moreover, a new, larger fusion plasmid was formed via IS*1216E-*mediated translocation, generated by the integration of an IS*1216E*-based transmissible unit into the mobilizable *poxtA*-carrying plasmid (30). In both cases, the fused *poxtA* plasmids could be mobilized with the aid of conjugative plasmids in the same strains.

Transmissible plasmids can be classified as conjugative or mobilizable plasmids. In order to realize the transfer of the mobilizable plasmids, at least two processes must be taken into account: (i) the mobilizable plasmids can be mobilized by functions encoded in *trans* provided by other auxiliary conjugative elements, where a distinct relaxase gene expressing a Mob protein on the majority of mobilizable plasmids targets an *oriT* and the resulting relaxosome is recruited to the T4SS encoded by a coresident conjugative element (27), as observed in our previous study (30); or (ii) the mobilizable plasmids could be directly fused to conjugative plasmids by IS-mediated replicative transposition or homologous recombination through an identical HR, which caused the mobilizable plasmids to become conjugative, as observed in this study.

Several studies have shown that replicative transposition or homologous recombination played an important role in the evolution of plasmid backbones and the acquisition of antimicrobial resistance genes in Gram-negative bacteria (28, 29, 31–33). To the best of our knowledge, this is the first time that the roles of replicative transposition and homologous recombination were shown in the shaping of *poxtA*-carrying plasmids from mobilizable to conjugative in enterococci, which are important health care-associated Gram-positive opportunistic pathogens. Because the mobilizable plasmids are not self-transmissible, their transmission usually requires the assistance of conjugative plasmids or other conjugative elements. In this study, replicative transposition and homologous recombination have been shown to participate in changing two mobilizable *poxtA*-carrying plasmids from nonconjugative to conjugative; as a result, these fused *poxtA*-carrying plasmids become self-transmissible, which accelerates their transmission.

Pheromone responsive plasmid transfer systems are highly efficient for genetic exchanges that allow antimicrobial resistance genes to be spread within bacterial populations (34). In our previous study, two pheromone-responsive conjugative *optrA*-carrying plasmids which showed high transfer frequencies of ∼10^−2^ were identified in porcine E. faecalis isolates (35). In a recent study, pheromone-responsive plasmids were proven to be the predominant plasmids in *optrA*-carrying E. faecalis clinical isolates, and most of them were conjugative (24). In this study, the conjugative *optrA*-carrying pheromone-responsive plasmid pE006-101 fused with the mobilizable *poxtA*-carrying plasmid, suggesting that linezolid resistance co-conferred by both *optrA* and *poxtA* genes in the same plasmid could be widely disseminated through sex pheromone plasmids.

In summary, this study showed that mobilizable *poxtA*-carrying plasmids can transfer through fusion into conjugative plasmids in enterococci. Two fusion events were identified: one through homologous recombination via an identical HR, the other through replicative transposition mediated by IS*1216E.* In both fusion events, the *poxtA*-carrying plasmids changed from mobilizable to conjugative, leading to the generation of new plasmids and the potentially enhanced dissemination of the *poxtA* gene in enterococci.

## MATERIALS AND METHODS

### Bacterial strains and antimicrobial susceptibility testing.

E. faecalis E006 and *E. lactis* E843 were isolated from fecal samples during a routine survey of antimicrobial resistance in bacteria from pigs in Henan Province, China, in 2018. Strain species of E843 and E006 were identified by PCR with 16s rRNA primers, followed by sequencing. E. faecalis JH2-2 (RIF^r^), E. faecium GE-1 (RIF^r^), and E. faecalis E533 (a strain without plasmids, identified and stored in our lab, TET^r^) served as recipient strains in transfer experiments. AST was performed using broth microdilution according to the recommendations given in the M100 (31st ed.) of the Clinical and Laboratory Standards Institute (CLSI; 36). S. aureus ATCC 29213 served as the quality control strain.

### PCR analysis and transfer experiments.

The presence of the resistance gene *poxtA* in E. faecalis E006 and *E. lactis* E843 was determined by PCR using the primers described previously (13). All PCR products were subjected to Sanger sequencing. Conjugation experiments were performed as previously described (37). In brief, the overnight cultures of donors and recipients were mixed at a ratio of 1:4 (200:800 μL), centrifuged, and suspended with 100 μL fresh brain heart infusion (BHI) broth; the mixed bacteria were then plated on BHI agar at 37°C for 18 h. The bacteria grown on the BHI agar were sluiced off with 4 mL BHI broth, and 100 μL of the culture was plated on selective BHI agar. When E. faecalis E006 was used as the donor, E. faecalis JH2-2 was used as the recipient; when *E. lactis* E843 was used as the donor, E. faecium GE-1 served as the recipient. Transconjugants were selected on BHI agar containing rifampicin (50 mg/L) and florfenicol (10 mg/L) and further confirmed by AST and multilocus sequence typing (MLST) following harmonized protocols (https://pubmlst.org/). The positive transconjugants were E. faecalis E006×JH2-2-TC1 and E. faecium E843×GE-1-TC1, respectively. To assess the self-transferability of the fusion plasmids, further conjugation experiments were performed. When E. faecalis E006×JH2-2-TC1 was used as the donor, E. faecalis E533 (TET^r^) was used as the recipient, and the transconjugants were selected on BHI agar containing tetracycline (10 mg/L) and florfenicol (10 mg/L). When E. faecium E843×GE-1-TC1 was used as the donor, E. faecalis JH2-2 [VAL^r^, natural resistance due to the presence of *lsa*(A)] served as the recipient, and the transconjugants were selected on BHI agar containing valnemulin (64 mg/L) and florfenicol (4 mg/L). All transconjugants were further confirmed using the methods described above.

### S1-PFGE and Southern blot hybridization.

The genomic DNA was digested with S1 endonuclease (New England Biolabs, Beverly, MA, USA) separated by PFGE as previously described (38). After transfer to Amersham Hybond-N^+^ membranes (GE Healthcare), the genomic DNA was hybridized with a *poxtA* probe as described in other studies (30).

### WGS and sequence analysis.

To investigate the genetic basis of plasmid alterations in transconjugants, whole-genome DNA of E. faecalis E006 and *E. lactis* E843, and their transconjugants, E006×JH2-2-TC1 and E843×GE-1-TC1, was sequenced using the Oxford Nanopore and Illumina MiSeq platforms (Shanghai Personal Biotechnology Co. Ltd., China). The Nanopore sequence reads were assembled with HGAP4 and CANU (version 1.6) and corrected by Illumina MiSeq with Pilon (version 1.22). The prediction of open reading frames and their annotation was performed using Glimmer 3.0. The plasmid replicon genotype was identified using PlasmidFinder (https://cge.cbs.dtu.dk/services/PlasmidFinder/). Insertion sequence (IS) elements were identified using ISfinder (https://isfinder.biotoul.fr/). Comparative analysis and plasmid maps were generated by Easyfig and BRIG (39, 40).

### Accession number(s).

The sequences of the six plasmids pE006-101, pE006-19, pE006-TC-121, pE843-171, pE843-27, and pE843-TC-200, determined in this study, have been deposited in GenBank under the accession numbers CP082232, CP082233, CP081506, CP082266, CP082268, and CP081503, respectively.
